# Dietary Polysaccharide from *Enteromorpha Clathrata* Modulates Gut Microbiota and Promotes the Growth of *Akkermansia muciniphila*, *Bifidobacterium* spp. and *Lactobacillus* spp.

**DOI:** 10.3390/md16050167

**Published:** 2018-05-17

**Authors:** Qingsen Shang, Ya Wang, Lin Pan, Qingfeng Niu, Chao Li, Hao Jiang, Chao Cai, Jiejie Hao, Guoyun Li, Guangli Yu

**Affiliations:** 1 Key Laboratory of Marine Drugs of Ministry of Education, and Shandong Provincial Key Laboratory of Glycoscience and Glycotechnology, School of Medicine and Pharmacy, Ocean University of China, Qingdao 266003, China; shangqingsen@163.com (Q.S.); 15222175867@163.com (Y.W.); pl_panlin@163.com (L.P.); qfengniu@163.com (Q.N.); lichaolbj@163.com(C.L.); haojiang833@163.com (H.J.); caic@ouc.edu.cn (C.C.); 2009haojie@ouc.edu.cn (J.H.); liguoyun@ouc.edu.cn (G.L.); 2 Laboratory for Marine Drugs and Bioproducts, Qingdao National Laboratory for Marine Science and Technology, Qingdao 266003, China

**Keywords:** *Enteromorpha clathrata*, polysaccharide, gut microbiota, prebiotic, *Bifidobacterium* spp., *Akkermansia muciniphila*, *Lactobacillus* spp.

## Abstract

Recently, accumulating evidence has suggested that *Enteromorpha clathrata* polysaccharide (ECP) could contribute to the treatment of diseases. However, as a promising candidate for marine drug development, although ECP has been extensively studied, less consideration has been given to exploring its effect on gut microbiota. In this light, given the critical role of gut microbiota in health and disease, we investigated here the effect of ECP on gut microbiota using 16S rRNA high-throughput sequencing. As revealed by bioinformatic analyses, ECP considerably changed the structure of the gut microbiota and significantly promoted the growth of probiotic bacteria in C57BL/6J mice. However, interestingly, ECP exerted different effects on male and female microbiota. In females, ECP increased the abundances of *Bifidobacterium* spp. and *Akkermansia muciniphila*, a next-generation probiotic bacterium, whereas in males, ECP increased the population of *Lactobacillus* spp. Moreover, by shaping a more balanced structure of the microbiota, ECP remarkably reduced the antigen load from the gut in females. Altogether, our study demonstrates for the first time a prebiotic effect of ECP on gut microbiota and forms the basis for the development of ECP as a novel gut microbiota modulator for health promotion and disease management.

## 1. Introduction

*Enteromorpha clathrata* is an edible green alga that has been traditionally consumed as a folk medicine and a natural herb in Asian countries for the treatment of inflammation-associated diseases [1,2]. Recently, a significant body of literature has demonstrated that polysaccharide from *E. clathrata* possesses numerous bioactivities including, but not limited to, anticoagulative [3], immunomodulatory [4], antioxidant [2,5], anticancer [6] and antiobesity [1,2]. Since seaweed polysaccharides are among the focus of marine biomedical research today [7,8], these discoveries make *E. clathrata* polysaccharide (ECP) an excellent candidate for marine drug development. However, to date, although different bioactivities of ECP have been studied, its effect on gut microbiota has not been explored.

Gut microbiota is an “invisible organ” that has been discovered to play pivotal roles in health and disease [9,10]. In recent years, it has become increasingly clear that disruption of the gut microbiota, which is commonly termed as “gut dysbiosis”, is robustly associated with various diseases and disorders such as diabetes [11], obesity [11,12], hypertension [13], colorectal cancer [14], multiple sclerosis [15], asthma [16], autism [17], Alzheimer’s disease [18], Parkinson’s disease [19] and non-alcoholic fatty liver disease [20]. Recent findings suggest an effective strategy for the management of diseases by targeting the gut microbiota [9,10,21], and among the commercially available therapeutics that are clinically used to treat dysbiosis and cure diseases, prebiotic stands out as a very effective one [22,23,24]. Previous studies have indicated that seaweed polysaccharides constitute an appreciable proportion of bioactive compounds for prebiotic development [25,26]. However, whether or not ECP has any prebiotic effects has not yet been determined. 

In the present study, the dearth of previous research, coupled with the fact that ECP cannot be absorbed after oral intake in the gastrointestinal tract [26,27], prompts us to investigate what impact ECP has on gut microbiota and whether it could be used as a prebiotic. To these ends, we treated male and female C57BL/6J mice with high (100 mg/kg/day) and low (50 mg/kg/day) doses of ECP and studied its effect on gut microbiota using 16S rRNA high-throughput sequencing. We found that ECP significantly changed the structure of the gut microbiota and exerted different prebiotic effects on *Akkermansia muciniphila*, *Bifidobacterium* spp. and *Lactobacillus* spp. in male and female mice. Our study provides the first proof-of-concept for the prebiotic effect of ECP and formed the basis for the development of ECP as a drug or a food supplement for health promotion and treatment of dysbiosis.

## 2. Results and Discussion

### 2.1. Dietary ECP Changed the Structure and Increased the Richness and Diversity of the Gut Microbiota in Male and Female Mice

**Figure 1:**
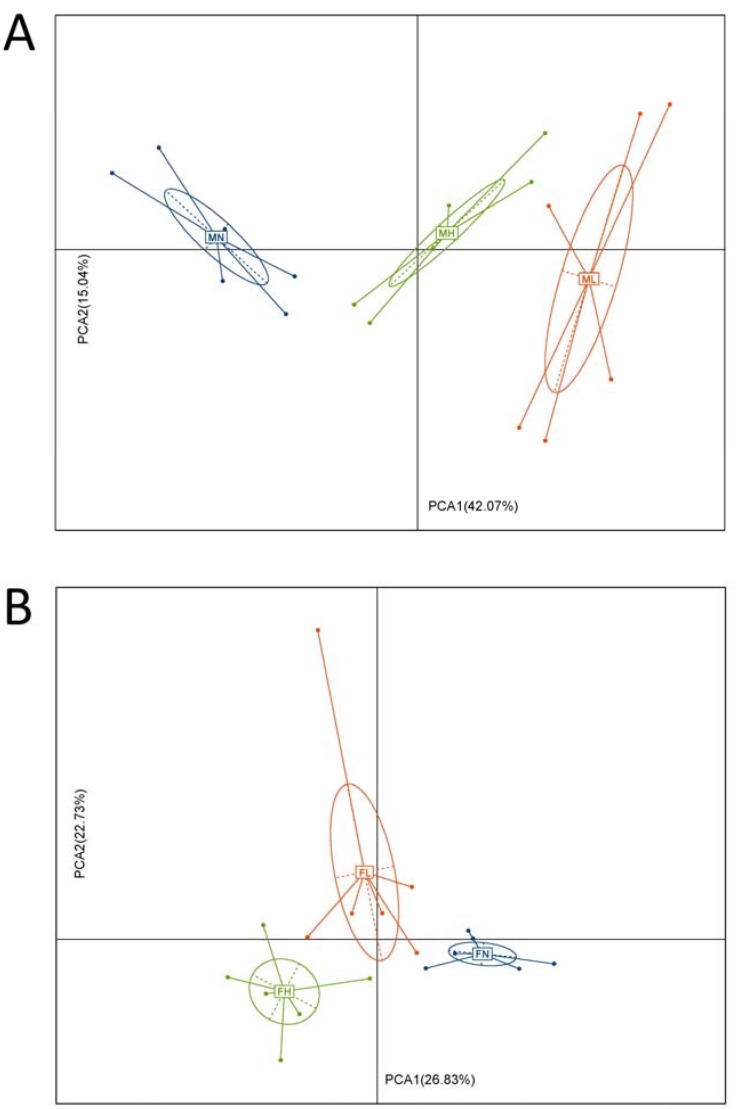
Administration of ECP significantly changed the structure of the gut microbiota. PCA score plot of the first and second components for the gut microbiome in male (**A**) and female mice (**B**). Blue indicates control groups, red indicates low dose groups and green indicates high dose groups. The PCA score plot was constructed based on the OTUs of the microbiota in male mice and female mice.

Previous studies indicate that ECP could be degraded by specific microbes in the gut [26,27], however, the precise effect of ECP on the whole gut microbiota is unknown. Therefore, to address this issue, we investigated here in detail the in vivo effect of ECP on intestinal microorganisms using 16S rRNA high-throughput sequencing. As revealed by principal component analysis (PCA), both low and high doses of ECP significantly changed the structure of the gut microbiota in C57BL/6J mice (Figure 1). Besides, alterations of the gut microbiota were more obvious in male mice than in female mice (Figure 1). This suggests that ECP has a sex-specific effect on gut microbiota. Interestingly, preceding studies have also found similar effects of chondroitin sulfate [28] and keratan sulfate [29] on gut microbiota. Given that sex hormones play a critical role in determining the composition of the intestinal microorganisms [30], our results and past studies [28,29] highlight that dietary fibers may have a gender-specific effect on gut microbiota and clinical therapies using dietary fibers for the treatment of dysbiosis-associated diseases should be tailored according to individuals’ sex. 

**Figure 2:**
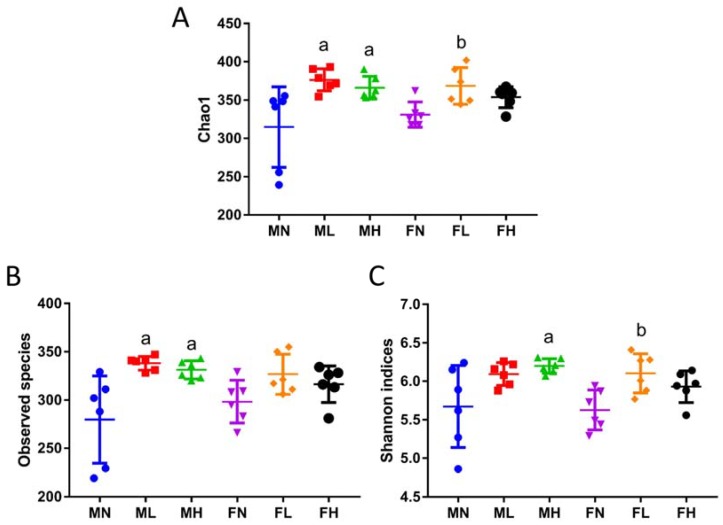
Dietary ECP increased the richness and diversity of the gut microbiota. Chao1 (**A**) and observed species (**B**) were used as the richness estimators. Shannon indices (**C**) was used as the diversity estimator. a: *p* < 0.05 vs. MN group; b: *p* < 0.05 vs. FN group.

Since ECP changed the structure of the gut microbiota, we then sought to investigate its effect on the richness and diversity of the intestinal microorganisms. To this aim, the values of the richness estimators, Chao1 and observed species, and the values of the diversity estimator, Shannon indices, were respectively calculated. Interestingly, ECP was found to significantly increase the richness and diversity of the gut microbiota in male and female mice (Figure 2). 

It is of interest to note that in Figure 1A, PCA1 and PCA2 explained about 60% of the variance for the male microbiota, while in Figure 1B, PCA1 and PCA2 only explained about 50% of the variance for the female microbiota. As have detailed above, the structure of the male microbiota is quite different from that of the female microbiota. In addition, previous studies have also demonstrated that sex hormones play a critical role in determining the composition of the gut microbiome [30]. Therefore, it is reasonable that the PCA plot of the female microbiota and male microbiota were different. Another reason for the dissimilarities of the PCA plot might be due to the fact that compared to the male microbiota, the female microbiota is more reluctant to ECP treatment. This means that ECP induces much less alterations in the composition of the female microbiota than that of male microbiota. In addition, this can be evidenced by the Chao1 indices, observed species and Shannon indices. As shown in Figure 2, ECP treatment resulted in a much more dramatic increase in Chao1 indices, observed species and Shannon indices in the microbiota of male mice, while in contrast, in female mice, only the microbiota from the low-dosage group seemed to have increased values of Chao1 indices and Shannon indices. This is in quite accordance with the PCA plot analysis. In male microbiota, both low-dosage ECP and high-dosage ECP significantly changed the structure of the microbiota along the PCA1 axis, which explained 42.07% of the variance (Figure 1A). However, in female microbiota, only the low-dosage group had the most obvious alterations in the composition of the microbiota as it changed both along the PCA1 axis (explained 26.83% of the variance) and the PCA2 axis (explained 22.73% of the variance) (Figure 1B). Although treatment of high-dosage ECP also modulated the composition of the microbiota in the FH group, it changed largely only along the PCA1 axis (explained 26.83% of the variance). This also explains why the compositions of the microbiota in FN group and FH group are closely related in comparison to the FL group.

Previous studies indicate that a fiber-deficient diet could decrease the richness and diversity of the gut microbiome and increase the susceptibility of the host to pathogen infection and intestinal diseases [31]. Besides, mounting evidence suggests that supplementation of dietary fiber or microbiota-accessible carbohydrate to the daily diet could help to maintain intestinal symbiosis and reduce the incidence of bacterial colonization [32,33]. In line with these results, our study demonstrated that ECP as a dietary fiber could modify the composition of the gut microbiota and increase species diversity and richness. Our findings support the use of ECP as a novel gut microbiota modulator and encourage continued investigation on the beneficial effect of ECP on gut microbiota in mouse models of dysbiosis-associated diseases.

### 2.2. Dietary ECP Modulated the Intestinal Microbiota at Different Taxonomic Levels and Proundly Increased the Populations of Akkermansia muciniphila, Bifidobacterium spp. and Lactobacillus spp.

**Figure 3:**
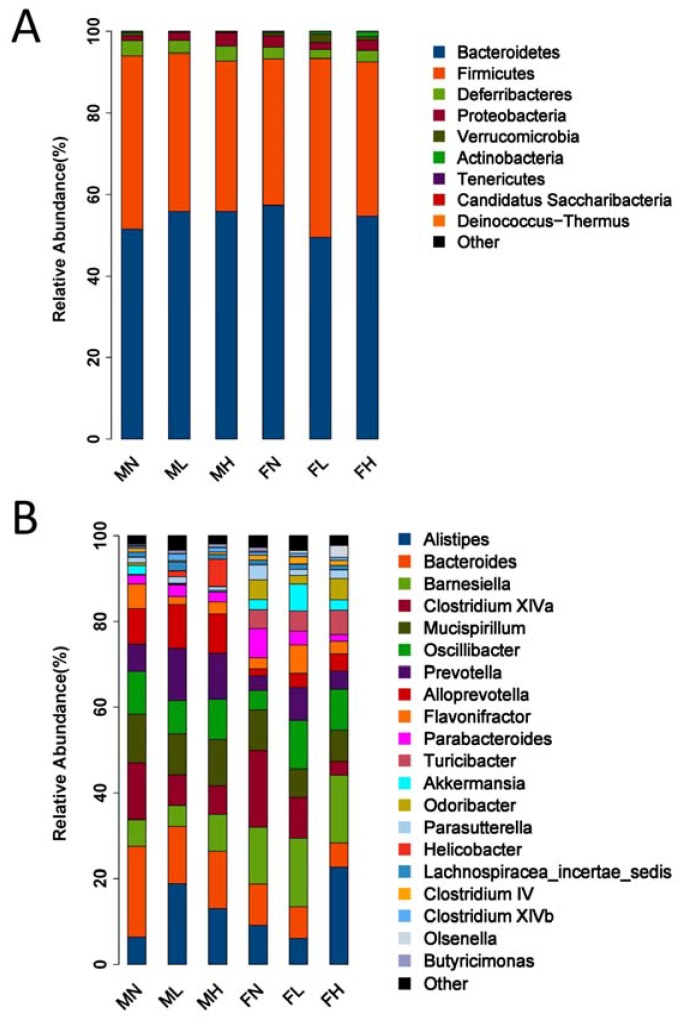
Response of the gut microbiota to ECP treatment at the phylum (**A**) and genus (**B**) levels. The relative abundances of the gut bacteria presented here were calculated by averaging the data obtained from the six replicates within each group.

Given that ECP could change the structure of the microbiota, we next asked what impact ECP has on the abundance of each bacterium in the gut. We compared the bacterial composition of the gut microbiota at the phylum level and found that ECP significantly increased the abundance of Bacteroidetes and decreased the population of Firmicutes in male mice (Figure 3A). However, interestingly, in female mice, ECP decreased the amount of Bacteroidetes and increased the proportion of Firmicutes (Figure 3A). Besides, ECP was also found to has a differing effect on the abundance of Proteobacteria in male and female mice. Male mice treated with ECP had increased levels of Proteobacteria while female mice fed with ECP had reduced levels of Proteobacteria (Figure 3A).

We further compared the bacterial composition of the intestinal microbiota at the genus level and found similar divergent effects of ECP on the abundances of *Alistipes* spp., *Oscillibacter* spp., *Flavonifractor* spp., *Parabacteroides* spp. and *Akkermansia* spp. in male and female mice (Figure 3B). For example, in male mice, ECP decreased the population of *Oscillibacter* spp., while in contrast, in female mice, ECP increased that of *Oscillibacter* spp. Collectively, these findings demonstrate that the male microbiota and female microbiota respond oppositely to ECP treatment, which, in accordance with the PCA results, provides a further proof of the sex-specific effect of ECP on gut microbiome.

**Figure 4:**
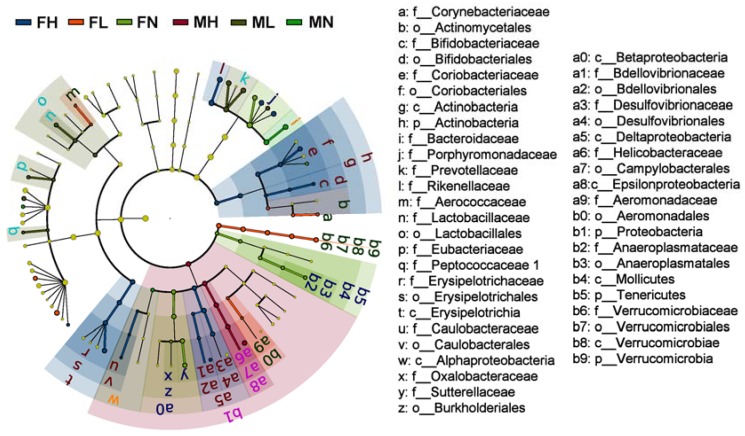
The taxonomic cladogram obtained from LEfSe analysis of gut microbiota in different groups. The microbial compositions of the male and female mice were compared at different evolutionary levels. A significant value of less than 0.05 was used as a threshold for the LEfSe analysis.

**Figure 5:**
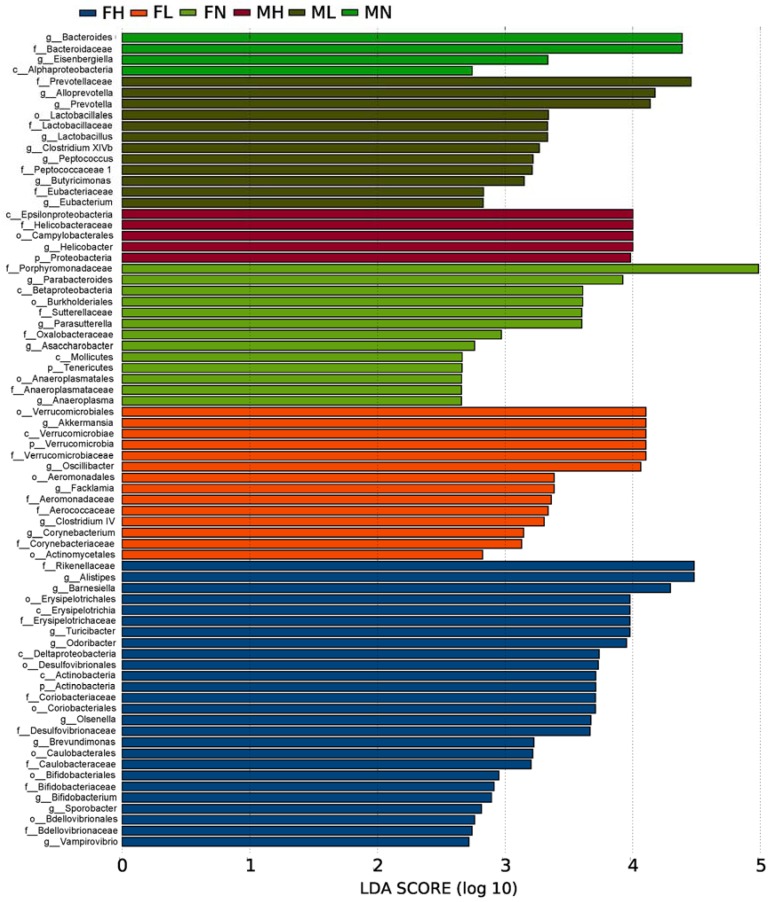
The LDA score obtained from LEfSe analysis of gut microbiota in different groups. A LDA effect size of more than 2 was used as a threshold for the LEfSe analysis.

To fully elucidate the effect of ECP on gut microbiota, we performed the linear discriminant analysis (LDA) effect size (LEfSe) analysis. The taxonomic cladogram and LDA score obtained from LEfSe analysis confirmed and visualized the modulatory effect of ECP on gut microbiota (Figure 4 and Figure 5). Briefly, in male mice, ECP increased the abundances of *Bacteroides* spp., *Prevotella* spp., *Alloprevotella* spp., *Butyricimonas* spp., *Eubacterium* spp., and *Peptococcus* spp. and decreased the proportion of *Helicobacter* spp., while in contrast, in female mice, ECP increased the abundances of *Odoribacter* spp. *Clostridium IV*, *Oscillibacter* spp. and *Alistipes* spp. and decreased the proportions of Betaproteobacteria and *Parabacteroides* spp. (Figure 4 and Figure 5). These observations are consistent with the sex-dependent effect of ECP on gut microbiota. 

**Figure 6:**
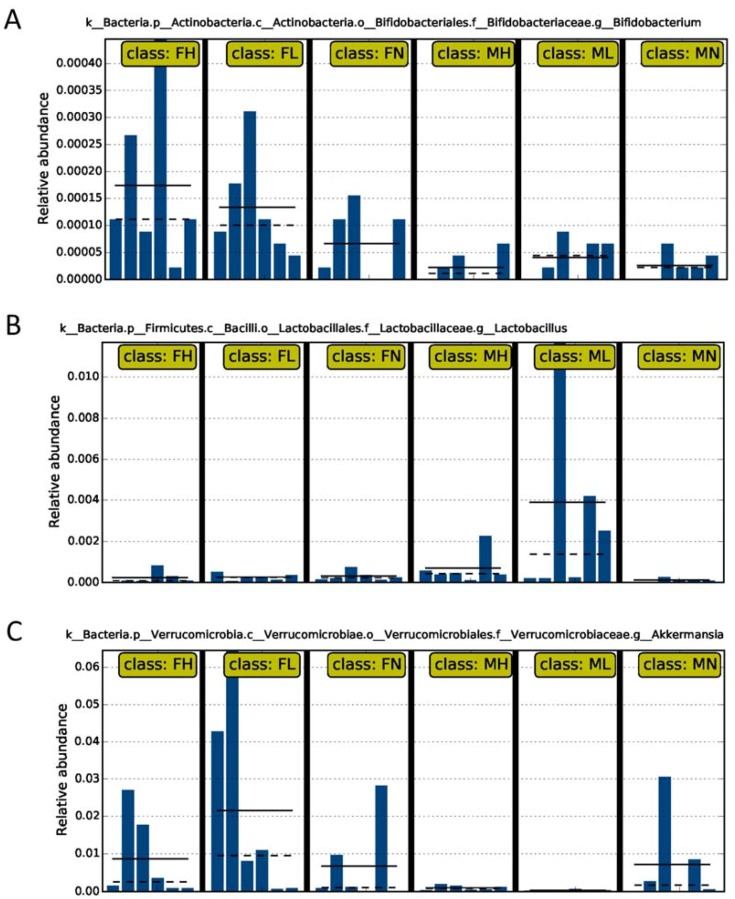
Intake of ECP significantly increased the abundances of *Bifidobacterium* spp. (**A**), *Lactobacillus* spp. (**B**) and *A. muciniphila* (**C**) in C57BL/6J mice. The solid line represents the average abundances of *Bifidobacterium* spp. (**A**), *Lactobacillus* spp. (**B**) and *A. muciniphila* (**C**) of the six replicates within each group; the dash line represents the median abundances of *Bifidobacterium* spp. (**A**), *Lactobacillus* spp. (**B**) and *A. muciniphila* (**C**) of the six replicates within each group. The differences in the abundances of the probiotic bacteria between treated mice and control mice were evidenced to be significant at *p* < 0.05 by LEfSe analysis.

Intriguingly, LEfSe analysis also identified that the abundances of the probiotic bacteria, including *Lactobacillus* spp., *Bifidobacterium* spp. and *Akkermansia* spp., were significantly different between the ECP-treated groups and control groups (Figure 4 and Figure 5). By comparing in detail the populations of the probiotic bacteria in all the six groups, we found that ECP significantly increased the abundances of *Bifidobacterium* spp. and *Akkermansia* spp. in female mice and that of *Lactobacillus* spp. in male mice (Figure 6). Specifically, ECP increased the amount of *Lactobacillus* spp. by about 35-fold in the ML group and by about 8-fold in the MH group (Figure 6). Similarly, ECP increased the amount of *Bifidobacterium* spp. by about 2-fold in the FL group and by about 3-fold in the FH group (Figure 6). Besides, in the FL group, ECP also significantly increased the population of *Akkermansia* spp. by about 3-fold (Figure 6). Collectively, these observations demonstrated a beneficial prebiotic effect of ECP on the gut microbiota in male and female mice.

Short chain fatty acids (SCFAs) are a class of critically important bacterial metabolites, which are produced by specific intestinal bacteria during fermentation of dietary fibers in the gut [34,35,36]. Accumulating evidence from animal and human studies suggests that SCFAs play a beneficial role in modulating host physiology, and increasing the concentrations of SCFAs in the gut contributes to the treatment of dysbiosis-associated diseases [37,38,39]. Here we discovered that the abundances of SCFA-producing bacteria [34,35,36], including *Bacteroides* spp., *Prevotella* spp., *Alloprevotella* spp., *Butyricimonas* spp., *Eubacterium* spp., *Odoribacter* spp. and *Clostridium IV* were significantly increased in the gut microbiota of male and female mice. Given the critical role of SCFAs in modulating host metabolism and physiology [37,38,39], these data suggest a favorable effect of ECP on the gut microbiota of C57BL/6J mice. 

It is of great interest to note that ECP remarkably increased the populations of *Akkermansia muciniphila*, *Bifidobacterium* spp. and *Lactobacillus* spp. in the gut (Figures 6). *A. muciniphila* is the only member of the genus *Akkermansia* spp. and the phylum Verrucomicrobia [40,41,42]. We and others have demonstrated that as an anti-inflammatory bacterium, *A. muciniphila* is inversely related to dysbiosis-associated diseases such as diabetes [43], obesity [44], metabolic syndrome [45] and colitis [46]. Recently, a large body of evidence has suggested the use of *A. muciniphila* for the treatment of metabolic disorders and gut diseases that are characterized with low-grade inflammation [40,41,42,45]. As such, *A. muciniphila* is now being widely developed as a next-generation probiotic and strategies to increase the abundance of *A. muciniphila* are currently a hot topic in this field [40,47]. *Bifidobacterium* spp. and *Lactobacillus* spp. are two common probiotics that have been extensively used in the food and pharmaceutical industries [48,49,50]. In addition, similar with that of *A. muciniphila, Bifidobacterium* spp. and *Lactobacillus* spp. have also been illustrated to be beneficial on a wide range of dysbiosis-associated diseases [51,52,53,54,55]. Here we demonstrate that ECP could significantly increase the abundances of *A. muciniphila* (by 3-fold), *Bifidobacterium* spp. (by 3-fold) and *Lactobacillus* spp. (by 35-fold) in C57BL/6J mice (Figure 6). However, it should be noted that ECP has a sex-specific effect on gut microbiota and, thus, the prebiotic effects on *A. muciniphila* and *Bifidobacterium* spp. were only found in female mice while that on *Lactobacillus* spp. were only found in male mice. Altogether, our data provides the first proof-of-concept for the use of ECP as a novel prebiotic for the promotion of health and management of dysbiosis-associated diseases. 

### 2.3. Dietary ECP Rduced the Antigen Load from the Gut in Female Mice and Maintained Normal Gut Homeostasis in Male Mice

**Figure 7:**
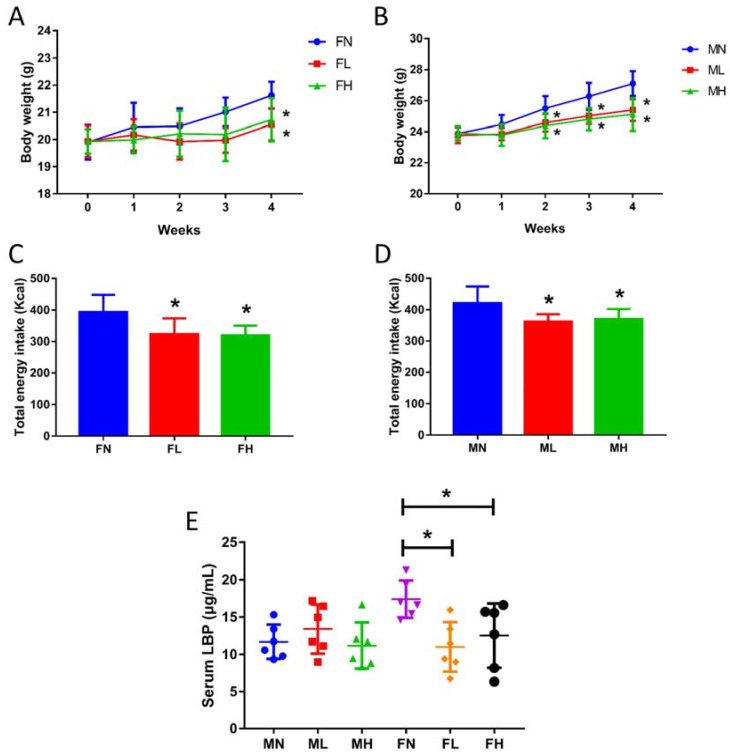
ECP treatment reduced body weight (**A**,**B**), energy intake (**C**,**D**) and serum LBP levels (**E**) in C57BL/6J mice. * *p* < 0.05 vs. the control group.

Previous studies indicate that dietary fibers from marine seaweeds with a prebiotic effect could decrease the body weight of experimental mice [29,56]. In accordance with previous reports, ECP, a dietary fiber, was also found to decrease the body weight and energy intake in male and female mice (Figure 7). Given that elevated concentrations of SCFAs could promote satiety and regulate energy homeostasis, the body weight-reducing effect of ECP may be resulted from the fact that ECP stimulates the growth of SCFA-producing bacteria and increases intestinal levels of SCFAs in the gut.

Lipopolysaccharide (LPS)-binding protein (LBP) is a well-established biomarker for studying the antigen load from the gut [57,58]. As an acute phase protein, it specifically binds to LPSs from the Gram-negative bacteria and antigens from the Gram-positive bacteria [57,58]. Therefore, to give an overall evaluation of the effect of ECP on gut microbiota, we further determined the serum LBP levels of male and female mice in different groups. Significantly, we found that ECP remarkably decreased serum LBP levels in female mice (Figure 7). This indicates that ECP could help to maintain a more balanced structure of the gut microbiota in female mice. However, interestingly, we did not detect any changes in serum LBP levels in male mice, suggesting that ECP had no toxic effect on the microbiome and could help to maintain normal gut homeostasis. As have detailed above, ECP has a differing effect on male and female microbiota. In this light, the discrepancy of serum LBP response to ECP treatment between male and female mice may be related to the sex-specific effect of ECP on gut microbiota. 

Our study has two limitations. First, organ blood flow plays a pivotal role in maintaining the colonic mucosal integrity [59]. In addition, previous studies have demonstrated beneficial impacts of microcirculation on protection and healing of the mucosa in the gastrointestinal tract [60,61]. Ghrelin and obestatin are two important peptides with a multiplicity of physiological functions [62,63]. In the intestine, ghrelin and obestatin exert fundamental effects on colonic homeostasis, food intake, immune function and glucose and energy metabolism by regulating microcirculation [64,65,66,67,68]. In this sense, given that ECP significantly modulated the composition of the gut microbiota and reduced the food intake of C57BL/6J mice, it is highly possible that ECP could also have changed the serum levels of ghrelin and obestatin. However, due to the experimental design, we only focused on the effect of ECP on gut microbiota and as such, we did not determine the concentrations of these two bioactive peptides in the microcirculation system of the intestine. Therefore, we anticipate that future studies could provide more information on this topic. Second, it has been demonstrated that an increase of the probiotic bacteria, including *A. muciniphila*, *Bifidobacterium* spp. and *Lactobacillus* spp., could change the structure of the mucus layer in the colon [40,41,42,43,44,45,46,47,51,52,53,54,55]. However, as we primarily focused on the modulatory effect of ECP on the gut microbiome, we did not conduct any histological tests of the colon tissues. Therefore, further studies are encouraged to address this issue.

Altogether, our study demonstrates for the first time a favorable effect of ECP on gut microbiota by promoting the growth of beneficial microbes and forms the basis for the development of ECP as a novel prebiotic for health promotion and disease management.

## 3. Materials and Methods

### 3.1. Chemicals and Reagents

ECP was isolated and purified from *Enteromorpha clathrata* (sourced from Qingdao, China) and was obtained from Qingdao Seawin Biotech Group (Qingdao, China). The chemical structures of ECP were determined applying the protocols described elsewhere [45,46,47,48,49,50,51,52,53,54,55,56]. The molecular weight and sulfate content of ECP were found to be 11.67 kDa and 14.7%. The monosaccharide composition of ECP was determined as Man:Rha:GlcA:Glc:Gal:Xyl = 1.0%:49.7%:10.8%:29.9%:1.3%:7.2%. All other chemicals used were at analytical grade and were obtained from Sigma (Shanghai, China). 

### 3.2. Animals and Treatment

All the animal experiments were approved by the Ethical Committee of Ocean University of China (Permission No. OUC-2017-0509-01). Briefly, a total of 36 specific pathogen-free C57BL/6J mice (six-week old, 18 males and 18 females) were purchased from the Vital River Laboratory Animal Technology Co. Ltd. (Beijing, China) (Certificate No. SCXK (Jing) 2016-0011). All mice were housed in a well-controlled environment (22–23 °C, 12 h light/dark cycles). After a three-week adaptation period, all animals were randomly allocated into 6 test groups: male control group (MN), male low-dosage group (ML), male high-dosage group (MH), female control group (FN), female low-dosage group (FL) and female high-dosage group (FH). The low-dosage groups were given ECP at 50mg/kg/day while the high-dosage groups were given ECP at 100 mg/kg/day. ECP was given by gavage and the control groups were given equal volume of normal saline. After 4 weeks of treatment, all mice of the 6 groups were sacrificed by cervical dislocation. Blood samples were collected from the orbital plexus and centrifuged at 1200 rpm for 20 min to pellet the blood cells. The serum LBP levels were determined using a commercial ELISA kit (Cell Sciences, Canton, MA, USA). The cecum was harvested and the cecal content in each mouse was aseptically collected and stored at −80 °C before being analyzed.

### 3.3. DNA Preparation and 16S rRNA High-throughput Sequencing

The metagenomic DNA was extracted from the cecal content using a QIAamp DNA Stool Mini Kit (Qiagen, Hamburg, Germany). The concentrations of the obtained DNA were determined using a Nanodrop ND2000 UV-vis spectrophotometer (Thermo Scientific, Wilmington, NC USA). After checking the DNA quality by gel electrophoresis, a pair of universal primers (341F 5’-CCTACGGGRSGCAGCAG-3’ and 806R 5’-GGACTACCAGGGTATCTAAT-3’) were then applied to specifically amplify the V3-V4 hypervariable regions of the 16S rRNA gene using the method previously described [45,56]. The amplicons were purified and quantified before being sequenced in the HiSeq 2500 platform (Illumina, San Diego, CA USA) from a commercial company (Realbio Technology Co. Ltd., Shanghai, China).

### 3.4. Bioinformatics and Sequencing Data Analysis

USEARCH was applied to filter the chimeras and singletons of the raw sequencing data and to cluster the high-quality reads into operational taxonomic units (OTUs) at the 97% similarity level. After that, a representative sequence of each of the obtained OTUs was assigned to a taxonomic level in the Ribosomal Database Project (RDP) database using the RDP taxonomic nomenclature and the RDP classifier. To evaluate the community diversity and community richness, the values of Chao1, observed species and Shannon indices were calculated using Mothur (version V.1.30.1, University of Michigan, Ann Arbor, MI, USA). Principal component analysis (PCA) of the OTUs in different groups was conducted using R packages (version 3.1.0, R Foundation for Statistical Computing, Vienna, Austria) to compare the structure of the gut microbiota of male and female mice [45,46,47,48,49,50,51,52,53,54,55,56]. LEfSe analysis coupled with the Kruskal-Wallis rank sum test was performed to identify the microbial differences among all the groups [45,46,47,48,49,50,51,52,53,54,55,56]. The LDA score for each bacterium was calculated and a taxonomic cladogram was constructed to visualize the differences of the microbial composition. A significant value of less than 0.05 and a LDA effect size of more than 2 was used as thresholds for the LEfSe analysis. 

### 3.5. Statistical Analysis

Data for Chao1, observed species, Shannon indices, body weight, total energy intake and serum LBP levels were expressed as mean ± standard deviation. Statistical analyses of the above data were performed by one-way ANOVA followed by Dunnett’s test (GraphPad Prism 6.00, La Jolla, CA, USA). All results were considered statistically significant at *p* < 0.05 versus the control group.
